# Transmission Properties of FeCl_3_-Intercalated Graphene and WS_2_ Thin Films for Terahertz Time-Domain Spectroscopy Applications

**DOI:** 10.1186/s11671-019-3062-3

**Published:** 2019-07-09

**Authors:** Maria O. Zhukova, Benjamin T. Hogan, Egor N. Oparin, Polina S. Shaban, Yaroslav V. Grachev, Evgeniya Kovalska, Kieran K. Walsh, Monica F. Craciun, Anna Baldycheva, Anton N. Tcypkin

**Affiliations:** 1 Laboratory of Femtosecond Optics and Femtotechnology, ITMO University, St. Petersburg, Russia; 2 EPSRC Centre for Doctoral Training in Metamaterials, University of Exeter, Exeter, UK

**Keywords:** Graphene, Layered materials, Tungsten disulfide, Liquid crystals, Terahertz radiation, Spectroscopy

## Abstract

Time-resolved terahertz spectroscopy has become a common method both for fundamental and applied studies focused on improving the quality of human life. However, the issue of finding materials applicable in these systems is still relevant. One of the appropriate solution is 2D materials. Here, we demonstrate the transmission properties of unique graphene-based structures with iron trichloride FeCl_3_ dopant on glass, sapphire and Kapton polyimide film substrates that previously were not investigated in the framework of the above-described problems in near infrared and THz ranges. We also show properties of a thin tungsten disulfide WS_2_ film fabricated from liquid crystal solutions transferred to a polyimide and polyethylene terephthalate substrates. The introduction of impurities, the selection of structural dimensions and the use of an appropriate substrate for modified 2D layered materials allow to control the transmission of samples for both the terahertz and infrared ranges, which can be used for creation of effective modulators and components for THz spectroscopy systems.

**Electronic supplementary material:**

The online version of this article (10.1186/s11671-019-3062-3) contains supplementary material, which is available to authorized users.

## Introduction

The field of terahertz time-domain broadband spectroscopy based on femtosecond near-infrared lasers has become an active research area due to its prospective application in non-destructive control [1], biomedicine [2], security systems, broadband communications [3] and others [4]. Despite the promise for applications and observed use of the technology in both industry and scientific projects, there is still a marked lack of effective materials for generation, detection, filtering and modulation of THz radiation. Solid materials applicable for THz time-domain spectroscopy systems (THz-TDS) can be classified into several groups: nonlinear and semiconductor crystals, organic crystals and metamaterials, composites, and 2D materials. 2D materials present a promising solution due to their compact size and the additional possibility to control the properties by modifying the number and composition of layers, and the substrate type.

Layered materials that can be exfoliated to extract individual layers can be primarily grouped into three classes [5]: graphene and its derivatives, chalcogenides and oxides. Graphene [6–8], molybdenum disulfide (MoS_2_) [9, 10], bismuth selenide Bi_2_Se_3_ [11], tungsten diselenide (WSe_2_) [12], tungsten disulfide (WS_2_) [13] and different devices based on layered heterostructures combining multiple individual 2D materials [14–16] have already been shown to demonstrate unique and exciting properties in the THz frequency ranges. It should be mentioned that, for the purposes of THz-TDS, materials which are stable at room temperature are more appropriate, as such materials minimise the additional operational requirements being placed on the overall system. Graphene has been widely proposed for different component parts of THz-TDS systems, specifically as detectors [17], polarizers [6], modulators [18, 19] and waveguides [20] and as a high harmonic generation medium [21, 22]. Layered WS_2_ has also been demonstrated as a THz generator [23, 24], as a modulator based on individual nanosheets [25] or liquid-exfoliated multilayer nanosheets [13], and furthermore as a magnetically tuned modulator [26, 27].

Typically, 2D materials are transferred to and then supported on a substrate. As laser-induced generation and detection is used in THz-TDS systems; hence, a substrate’s properties should be investigated in both the infrared and broadband THz ranges in addition to the 2D materials’ properties. Substrate materials with high transparency in the near-infrared and broad THz frequency ranges are desirable. Materials such as silicon, high-density polyethylene, polytetrafluoroethylene (Teflon), cyclic olefin copolymer (Topas), polyimide (Kapton), polyethylene terephthalate (PET) and others [28] are typically used in THz-TDS as they fulfil the transparency requirements. However, each substrate has a unique influence on the properties of a 2D material supported on it [29]. The effect of the substrate and the 2D material on the overall properties of a device are intrinsically coupled. Also, the specific topography of the interface region can significantly affect the properties. Therefore, when studying new conformations of 2D materials in combination with different substrates, the overall effect should be taken into account.

In this work, we demonstrate the transmission properties of unique graphene-based structures intercalated with a FeCl_3_ dopant [30] on glass, sapphire and Kapton polyimide film substrates. This material has not previously been investigated in relation to the problems described above within the NIR and THz (0.1 – 2 THz) ranges. We also show properties of thin WS_2_ films fabricated from liquid crystalline (LC) solutions transferred to Kapton and PET substrates in the same electromagnetic ranges. The work shows that the introduction of dopant impurities, the selection of structural dimensions and the use of an appropriate substrate for 2D layered materials allows one to control the transmission of samples for both the terahertz and near-infrared ranges, which can then be used to create effective modulators and components for future THz spectroscopy systems.

## Experimental Methods

### Fabrication of Samples

**Fig. 1:**
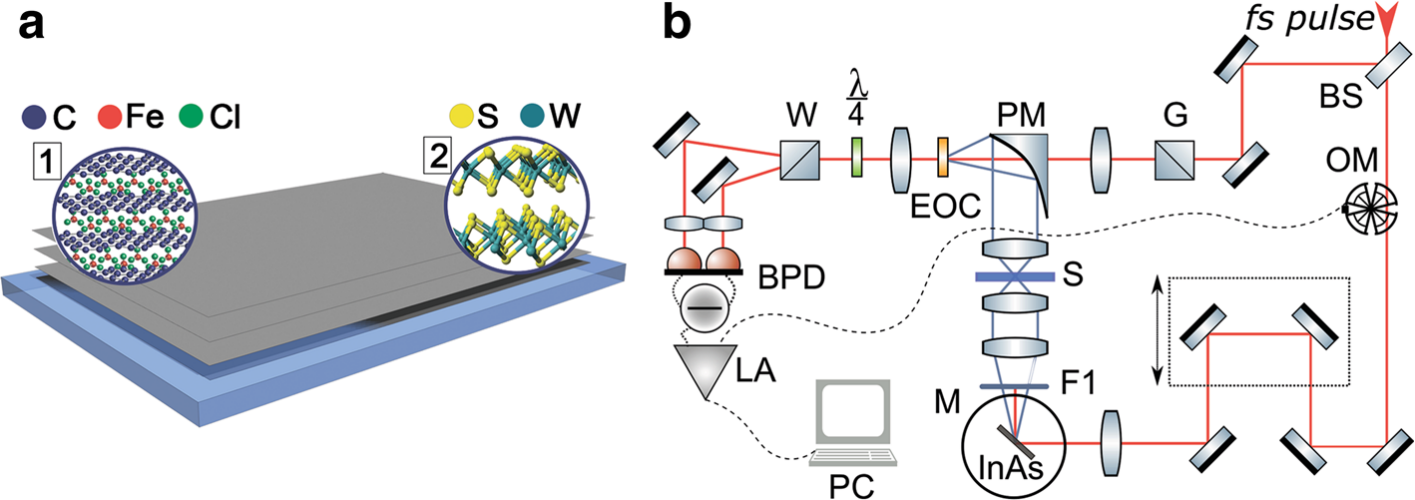
Structure of samples and experimental setup. **a** Representation of layered samples structure 1 - FeCl_3_ intercalated graphene layers, 2 - WS_2_ film fabricated from LC phase; **b** The laboratory terahertz time-domain spectrometer setup. fs pulse is divided by a beamsplitter (BS) to the pump and probe beams. The pump beam is modulated by an optical chopper (OM), passes delay line and is focused on InAs crystal in the magnet (M). A Teflon filter (F1) is used to cut off the IR pump beam. Generated THz pulses are focused on the sample (S) then collimated on the electro-optical crystal (EOC) by off-axis parabolic mirror (PM). The probe beam polarization is fixed by a Glan prism (G). The polarization change is registered by a quarter wave plate (*λ*/4), a Wollaston prism (W) and a balanced photo detector (BPD). Lock-in amplifier (LA) and personal computer (PC) are used for signal processing

Figure 1a illustrates the structures of the different layered samples discussed in this paper. The graphene-based samples (single layer- SLG; few-layer- FLG, 5-6 atomic layers; and multilayer graphene - MLG, 50–60 atomic layers) were synthesised on metallic (either copper or nickel) catalysts using a chemical vapour deposition (CVD) system and methane as a carbon source. The FLG and MLG samples were then intercalated (giving samples denoted i-FLG and i-MLG, respectively) with ferric chloride (FeCl_3_) vapours in a CVD system, using an established process within a three zone furnace [30–32]. The intercalated samples were transferred to glass, sapphire and Kapton substrates with 1 mm, 0.8 mm and 0.125 mm thicknesses, respectively. To achieve the transfer, first the intercalated graphene was coated with polymethylmethacrylate (PMMA). The metal catalyst was then etched using a concentrated ferric chloride solution to leave just the intercalated graphene on PMMA. This was then transferred to the required substrate, and the PMMA removed by dissolving in acetone. The resultant intercalated samples have been extensively characterised in previous work [30, 31, 33–42]. In particular, high resolution scanning electron microsopy of intercalated samples is shown in [41]. Further scanning electron microscopy (SEM) and atomic force microscopy (AFM) images of the samples are shown in Additional file 1: Figure S1.

WS_2_ films were fabricated from liquid crystalline tungsten disulfide dispersions. Films from LC phase solutions show higher homogeneity than those fabricated from non-LC dispersions [43–45]. To obtain a LC phase dispersion, an initial 500 mL solution was prepared in a sealed beaker. IPA was used as the solvent and bulk WS_2_ particles (Sigma-Aldrich 243639), with dimensions around a few microns on average as the solute at a concentration of 5 mg mL ^−1^. To break down the material, a process of ultrasonication in an ultrasonic bath (James Products 120 W High Power 2790 mL Ultrasonic Cleaner) filled with deionised water was used. Five hour-long periods, separated by 30 min each to prevent excessive heating of the solvent, were used to ensure sufficient exfoliation of the sample. The resultant dispersions were then put through a process of centrifugation for 10 min at 2000 rpm to remove residual bulk material and narrow the distribution of particle sizes present in the solution. After centrifugation, the solution was fractioned, with only the supernatant extracted, to ensure only suitably sized particles remained. The resultant solution was then dried under vacuum (∼ 0.1 atm) in a Schlenk line to fully remove the solvent, before being re-dispersed in IPA again at concentration of 1, 5 and 100 mg mL ^−1^. After re-dispersion, the solutions were again ultrasonicated (for a few minutes) to prevent any aggregated exfoliated particles remaining in the solutions. As the concentration is changed significantly following the centrifugation step, it is necessary to re-establish the concentration following that step. Re-dispersing allows for accurate knowledge of the concentrations of the solutions without affecting the properties of the dispersed 2D material particles. The tungsten disulfide dispersions of all concentrations showed a separation of phases as the volume fraction of the liquid crystal phase was less than 100%.

This solution was then transferred to Kapton and PET substrates with 0.125 and 1 mm thickness, respectively. These substrates were chosen due to their low absorption in the terahertz region from 0.1 to 2.0 THz. For transfer to Kapton, a drop casting method was used with the 100 mg mL ^−1^ dispersion. For the first sample (denoted WS_2_ S), 50 *μ*L of solution from the upper, lower concentration, non-LC phase fraction was drop cast directly onto the Kapton substrate and allowed to dry. For the second sample (WS_2_ L), 50 *μ*L of solution from the lower, higher concentration, LC phase fraction was used. Drop cast samples were dried on a hot plate at 70 ^*circ*^C for 5 min. In both cases, individual particle sizes were measured by atomic force microscopy and scanning electron microscopy, with average sizes determined as 2.5 *μ*m^2^ laterally and thickness of 3.9 nm. The difference was the significantly greater overall film thickness for the L sample versus the S sample, owing to the greater concentration of tungsten disulfide in the liquid crystal phase fraction. For transfer to PET, a thin film transfer method was used. First 20 mL of the liquid crystalline solution was filtered using a Büchner flaskunder vacuum—under vacuum—onto a nano-porous polytetrafluoroethylene membrane. The film on the membrane was then transferred to the substrate using a heat- and IPA-assisted method. The substrate was wetted slightly with IPA while heating to 70 ^*circ*^C on a hot plate. The membrane was quickly transferred onto the substrate, and as the IPA evaporated through the membrane, the thin film of tungsten disulfide was released from the membrane and hence transferred to the substrate after removal of the membrane. Two samples were produced—one from the 1 mg mL ^−1^ dispersion (WS_2__LC) and the other from the 5 mg mL ^−1^ dispersion (WS_2__HC). Again, average individual tungsten disulfide particle sizes were determined as 2.5 *μ*m^2^ laterally and thickness of 3.9 nm. The overall film thicknesses were determined to be approximately 1 and 10 *μ*m respectively. Figure 3 shows SEM and optical images of the WS_2_ samples. In both cases, the uniformity of the coverage is noticeable. From SEM analysis, it can be seen that the majority of the particles are well aligned with the substrate, although some (typically smaller) particles are aligned perpendicular to the substrate. This general alignment is expected when depositing thin films from LC dispersions [43–46].

### Raman Spectroscopy

Raman spectroscopy measurements were conducted using a Raman spectrometer (Renishaw) with linearly polarised incident light at a wavelength of 532 nm and approximate power of 0.1 mW. Spectra were gathered with an accumulation time of 10 s.

### Visible and IR Range Spectroscopy

Measurements of intercalated graphene samples’ and tungsten disulfide films’ transmission in the visible and near infrared ranges were carried out using a research-class spectrophotometer (Evolution-300). This spectrometer allows measurement of the transmittance in 190–1100 nm range with standard deviation of 10 measurements < 0.05 nm and photometric accuracy of 1%.

### Terahertz Spectroscopy

The transmission in the THz range was investigated by a laboratory THz time-domain spectroscopy system [47, 48] which is systematised in Fig. 1b. In this system, the generation of THz radiation is based on the optical rectification of femtosecond pulses in an InAs crystal located in a magnetic field [49]. Femtosecond laser radiation from a Yb-doped solid-state fs oscillator (central wavelength 1050 nm, duration 100 fs, pulse energy 70 nJ, repetition rate 70 MHz) is divided by a beamsplitter (BS) to the pump and probe beams. The pump beam—modulated by an optical chopper—passes through a delay line and is focused on the THz generator InAs crystal placed in the magnet (M) with 2.4 T field. A Teflon filter (F1) is used to cut off the IR pump beam. The THz radiation (estimated average power 30 *μ*W, FWHM ∼1.8 ps) is focused at normal incidence on the sample (S). The transmitted THz pulse is collimated by a [100]-oriented CdTe electro-optical crystal (EOC) for EO detection by an off-axis parabolic mirror (PM). The probe beam polarization is fixed by a Glan prism (G) to be 45^*circ*^ relative to the THz polarization. The probe beam is also focused onto the same spot of the CdTe crystal. The birefringence in the CdTe crystal induced by the electric field of the THz pulse changes the polarization of the probe beam. The polarization change is measured using a quarter wave plate (*λ*/4), a Wollaston prism (W) and a balanced photo detector (BPD). A lock-in amplification (LA) technique is used to raise the signal-to-noise ratio. The amplified signal is then transferred to the computer via an analogue-to-digital converter.

The THz-TDS measurements were performed several times at different points of the samples and the averaged values were taken. The beam size in this setup is around 3 mm. The integral transmittance of the sample surface was measured. The obtained time dependencies of the THz pulse electric field (wave forms) without samples presence, when passed though substrates, and when passed through films on substrates were used to calculate THz frequency-domain spectra by means of Fourier analysis. The transmitted amplitudes were then compared for different samples.

## Results and Discussions

**Fig. 2:**
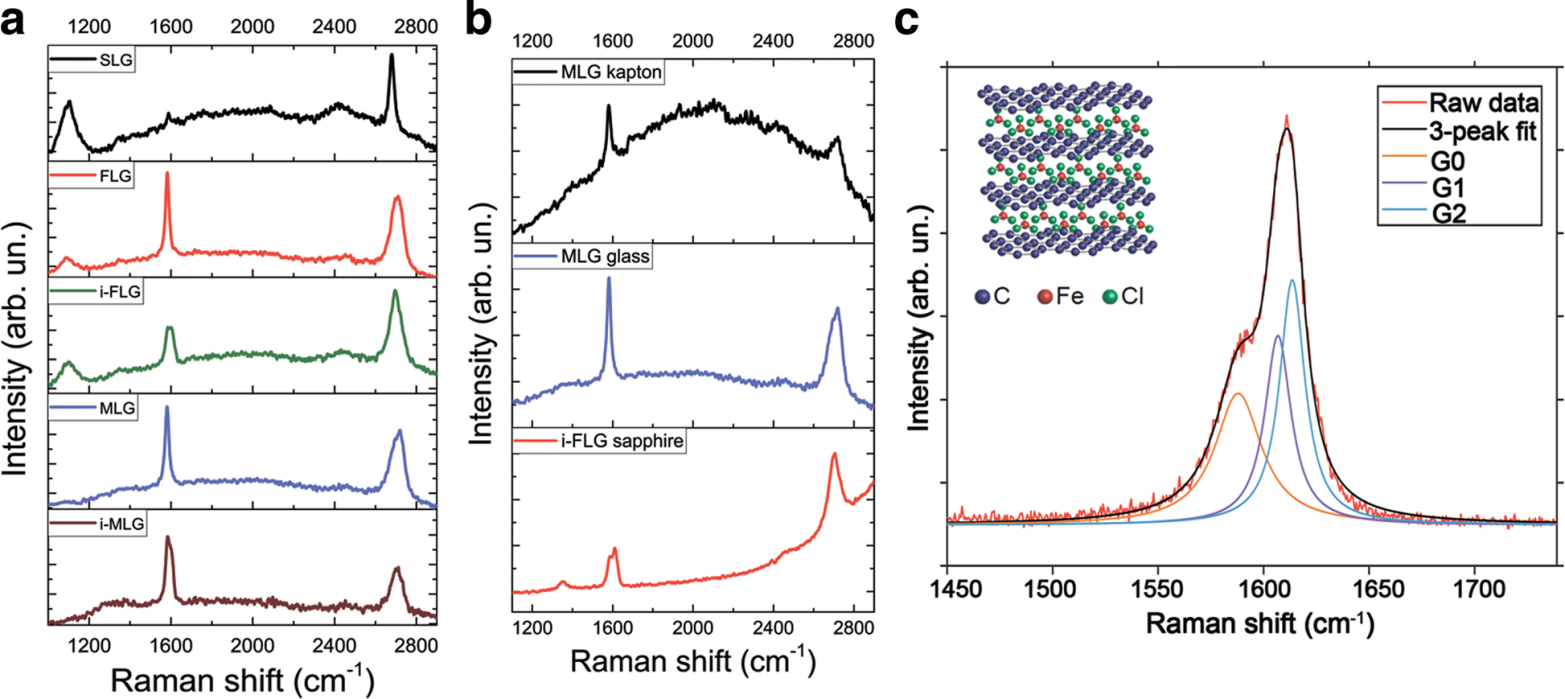
Raman spectra of graphene-based samples under study. Raman spectra of the different graphene samples on glass **a** and different substrates **b** performed using a 532-nm laser excitation system with a ×40 microscope objective, and 10 s integration time for a single scan. **c** shows the splitting of the *G* peak into 3 peaks in a i-FLG sample. As previously reported, the Raman shift of *G* to *G*0, *G*1 and *G*2 stem for a graphene sheet with randomly distributed FeCl_3_ molecules, one or two adjacent FeCl_3_ layers as shown by the schematic crystal structure

Raman spectroscopy can be used to determine the number of layers, the order in which layers are laid, orientation, doping, deformation and other properties of two-dimensional materials [50]. Raman spectra for graphene-based samples on glass (Fig. 2a) were taken and analysis of the main characteristic Raman modes (Additional file 1: Table S1) was performed. As seen in Fig. 2a for all kinds of graphene (SLG, FLG, MLG) on glass the location of the *G* peak varies slightly in the range 1582–1591 cm ^−1^. Whereas the 2*D* peak position of SLG in comparison to MLG undergoes a significant 41 cm ^−1^ upshift. Combined with the positions of the *G* and 2*D* peaks, the intensity ratio *I*_2*D*_/ *I*_*G*_ is determined by the number of layers and high quality of employed graphene samples. Additional peaks are observed for SLG, FLG and i-FLG on glass at around 1100 cm ^−1^. In fact, this behaviour is due to the increased influence of the glass substrate on the thinner, transparent structure of those graphene samples. Raman spectra for graphene-based samples on various substrates are shown in Fig. 2b and analysed (Additional file 1: Table S2). Typical graphene *G* and 2*D* peaks are observed for multilayer samples on Kapton (1579, 2721 cm ^−1^) and glass (1582, 2721 cm ^−1^) substrates, respectively. The influence of the substrate causes the shift of the main spectral features to higher wavenumbers [51, 52]. Meanwhile, the 2*D* peak (2703 cm ^−1^) and splitting of the *G* peak (1585, 1612, 1625 cm ^−1^) were observed for few-layer intercalated graphene on sapphire. The additional vibrational mode of *G* peak originates from the charge transfer from FeCl_3_ to graphene which results in an upshift of the *G*-band (Fig. 2c). The shift of the *G*-band to *G*1 = 1612 cm ^−1^ is a signature of a graphene sheet with only one adjacent FeCl_3_ layer, the shift to *G*2 = 1625 cm ^−1^ characterises a graphene sheet sandwiched between two FeCl_3_ layers, whereas randomly distributed FeCl_3_ dopants, impurities or surface charges give rise to the *G*0 peak with a Raman shift that varies between *G* in pristine graphene and *G*1 [30, 53]. The 2*D* peak for these samples is 18 cm ^−1^ downshifted. Such changes are caused by the smaller number of graphene layers, their structure and the influence of the intercalant. The intensity ratio *I*_2*D*_/ *I*_*G*_ for the samples is found to be equal to 0.8 (MLG on Kapton and glass) and 1.4 (i-FLG on sapphire). There is no evidence of the D peak for all analysed graphene samples, indicating high quality and stability of the sp^2^-hybridised carbon arrangement. The weak appearance of the *D* peak for i-FLG on sapphire (Fig. 2b) could be observed due to structural or edge defects occurring after intercalation. Thus, there is no significant substrate influence on the structural features of graphene of different nature.

**Fig. 3:**
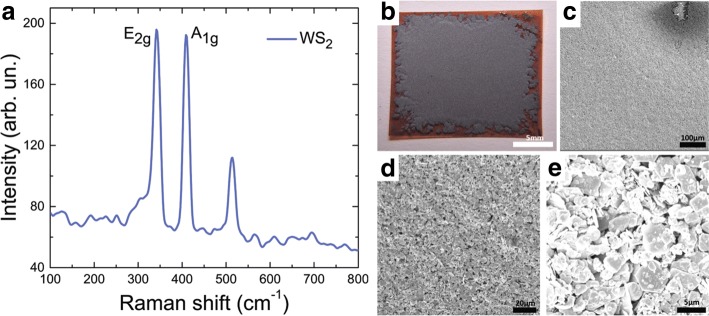
Raman spectra, photograph and SEM images of WS_2_ sample under study. **a** Raman spectrum of a few-layer WS_2_ film on silicon. **b** Photo of the drop cast film of WS_2_ on Kapton. **c**–**e** SEM images of the drop cast film of WS_2_ on Kapton at magnifications of **c** ×2000, **d** ×8000 and **e** ×40000

Figure 3a illustrates the Raman spectrum for tungsten disulfide film transferred from LC state to a silicon-on-insulator substrate. The typical peaks specific to crystalline WS_2_ *E*_2*g*_ and *A*_1*g*_ can be seen in the spectrum. Using Raman mapping for the thin films, high homogeneity of the Raman signal was observed over large areas.

**Fig. 4:**
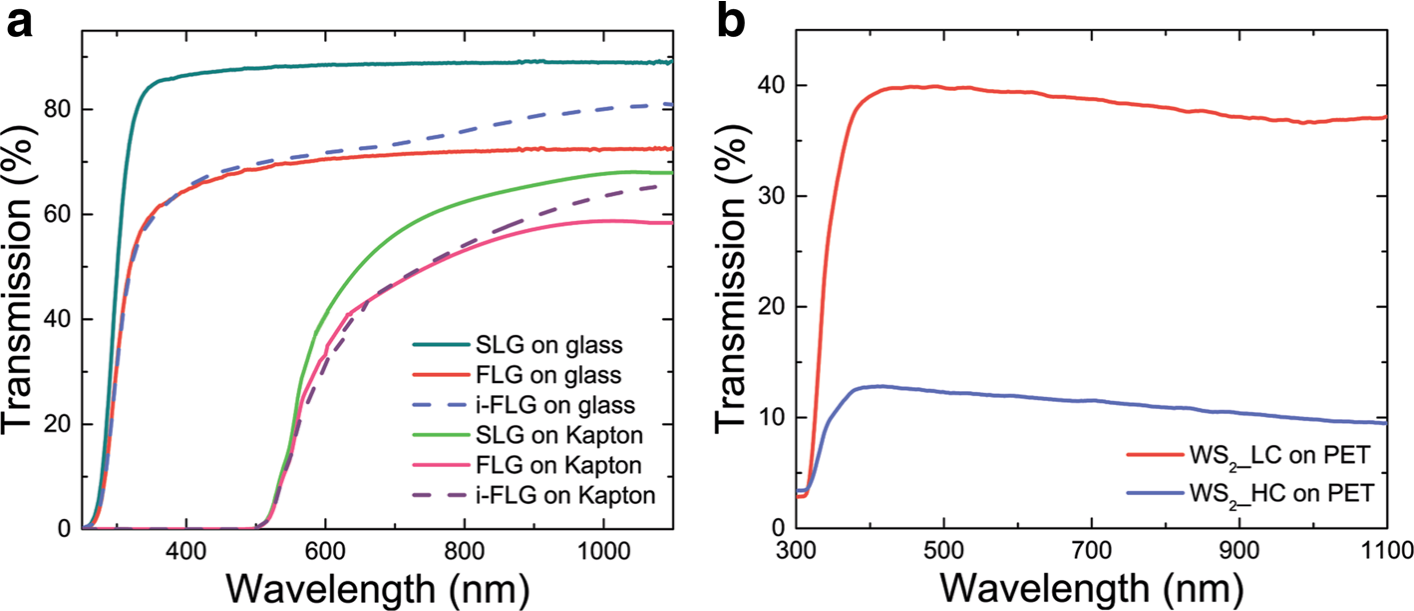
Transmission of the samples in visible and IR ranges. **a** Transmission of different amount of graphene layers on glass and Kapton substrates in UV-NIR range (SLG, MLG, i-MLG). **b** Transmission of WS_2_ film fabricated from LC phase solutions of different concentrations. WS_2__LC sample was produced from a 1 mg mL ^−1^ solution and WS_2__HC from a 5 mg mL ^−1^ solution

The transmission spectra in the visible-near infrared ranges of graphene-based and WS_2_ samples are shown in Fig. 4a and b, respectively. The achieved experimental information represents the integral transmittance of the samples. The scattering losses caused by the surface roughness are not separately evaluated; only the overall contribution of the sample to the transmitted radiation is taken into consideration. The intercalation of graphene leads to an increase of the sample transmission in the 700–1100 nm range. The increase can be explained by Pauli blocking occurring due to band filling [54, 55]. For example, at a wavelength of 1000 nm the transmittance of intercalated few-layer graphene (i-FLG) on glass is increased by 10%. This fact should be taken into account when using components based on intercalated graphene in THz-TDS systems, where they interact with both THz and IR radiation.

Varying the structure dimensions, specifically the film thickness, from 1 to 10 *μ*m for WS_2_ LC based thin films on polyethylene terephthalate (PET) causes a change of the transmission in the range from 400–1100 nm of up to 35%. This is expected due to the greater overall optical density of the thicker film produced from the higher concentration solution.

**Fig. 5:**
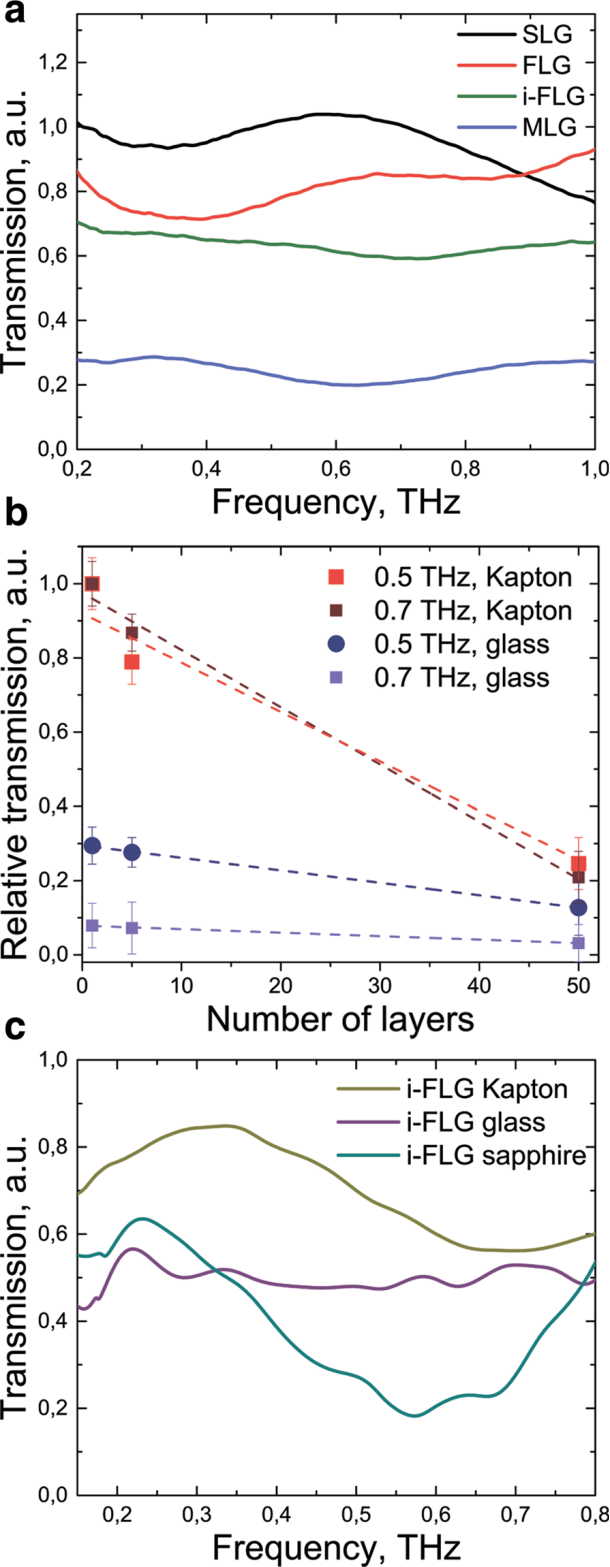
The experimental study of modified graphene samples by THz time-domain spectroscopy. **a** Transmission spectra of layered graphene in different modifications (SLG, single layer graphene, FLG few layer graphene, MLG multilayer graphene, i-FLG and i-MLG FeCl_3_ intercalated) on Kapton polyimide substrate. **b** The transmission as a function of graphene layer amount for 0.5 and 0.7 THz frequencies on Kapton and glass substrates. **c** Transmission of layered graphene relative to different substrates

Transmission spectra of broadband THz radiation (0.2–1 THz) through intrinsic and FeCl_3_ intercalated graphene-based samples on Kapton substrates are presented in Fig. 5a. In this case transmission spectra relative to air are presented. By increasing the number of layers, we can observe a decrease in the sample transmission for all substrates under study. This dependence of transmission as a function of layer number is linear for both different frequencies and different substrates (Fig. 5b) as was shown previously [37, 56]. This result shows that for pure graphene the increase of layer number does not change the material absorption coefficient in the THz frequency range (0.1–1 THz). To find the influence of FeCl_3_ intercalation, we observe the transmission relative to the substrate. Figure 5c shows transmission of intercalated few layered graphene (i-FLG) on glass, sapphire and Kapton substrates. The influence of intercalation and type of substrate can be seen in 0.4–0.8 THz range. It is demonstrated in relative enlightenment (for the case of polyimide up to 30%) and increasing of absorption (for the case of sapphire substrate up to 30%). It is highly likely that this changes are due to scattering by the graphene FeCl_3_ intercalated structure. In this case, the substrate affects the structure of the transferred material layers, and as a result, the THz radiation at different frequencies is scattered in different ways.

**Fig. 6:**
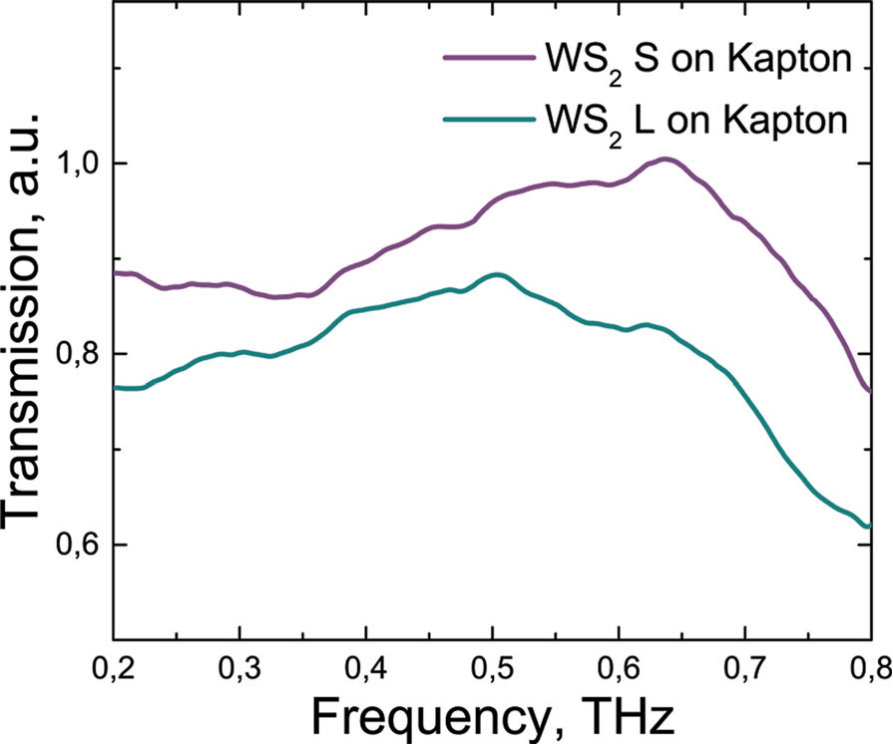
Transmission of WS_2_ samples in the THz frequency range. Spectra of WS_2_ films on Kapton substrates, produced from non-LC, low concentration fraction (WS_2_ S) and from LC phase, high concentration fraction (WS_2_ L)

WS_2_ on Kapton substrate, shown for different film thicknesses as described in the experimental methods, is fairly transparent in the THz range (Fig. 6). The transmission can be varied by choosing an appropriate concentration of the LC solution which is then transferred to substrate, and hence controlling the thickness of the drop-cast film. Transparency in the THz range is very useful for generation, detection and modulation applications for THz devices. It was shown [46] that for visible range such kind of liquid phase-exfoliated tungsten disulfide LC dispersions can demonstrate magnetically tuned dichroism in the liquid phase. The influence of magnetic part of electromagnetic field in THz range is more perceptible than in visible range, so it can be predicted, that the influence of THz magnetic field in such materials can be elucidated. It can be assumed that, with the help of WS_2_, it will be possible to control the magnetic field of THz pulse, as was shown in the concept of spin-current driven THz oscillator devices [57]. Such samples could also be used as magnetically tuned modulators in THz-TDS systems.

## Conclusions

In summary, the transmission properties of 2D layered materials based on graphene and tungsten disulfide in near infrared and terahertz ranges are demonstrated. Unique graphene-based structures intercalated with a FeCl_3_ dopant on glass, sapphire and Kapton polyimide substrates as well as thin WS_2_ film fabricated from liquid crystal solutions transferred to a Kapton and PET substrates were observed. The introduction of impurities, the intercalation, the selection of structural dimensions and the use of an appropriate substrate for modified 2D layered materials allow one to control the transmission of samples for both the terahertz and infrared ranges, which can be used for creation of effective modulators and components for THz spectroscopy systems. This work represents application-oriented results for future studies, which will concentrate on new devices for terahertz time-domain spectroscopy systems.

## Additional File

Additional file 1 Raman peak positions for different graphene samples on various substrates: SEM and AFM images of few-layer and intercalated few-layer graphene samples. (PDF 550 kb)

## Data Availability

The datasets used and analysed during the current study are available from the corresponding author on reasonable request.

## Abbreviations

AFM: Atomic force microscopy
CVD: Chemical vapour deposition
EO: Elecrto-optical
FLG: Few layer graphene
i-FLG: Intercalated few layer graphene
i-MLG: Intercalated multilayer graphene
i-SLG: Intercalated single layer graphene
IPA: Isopropanol
LC: Liquid crystal
MLG: Multilayer graphene
PET: Polyethylene terephthalate
PMMA: Polymethyl methacrylate
SEM: Scanning electron microscopy
SLG: Single layer graphene
THz-TDS: Terahertz time-domain spectroscopy

## References

[1] Dhillon SS, Vitiello MS, Linfield EH, Davies AG, Hoffmann MC, Booske J, Paoloni C, Gensch M, Weightman P, Williams GP (2017). The 2017 terahertz science and technology roadmap. J Phys D Appl Phys.

[2] Smolyanskaya OA, Schelkanova IJ, Kulya MS, Odlyanitskiy EL, Goryachev IS, Tcypkin AN, Grachev YV, Toropova YG, Tuchin VV (2018). Glycerol dehydration of native and diabetic animal tissues studied by THz-TDS and NMR methods. Biomed Opt Express.

[3] Grachev YV, Liu X, Putilin SE, Tsypkin AN, Bespalov VG, Kozlov SA, Zhang X-C (2018). Wireless Data Transmission Method Using Pulsed THz Sliced Spectral Supercontinuum. IEEE Photon Technol Lett.

[4] Mittleman DM (2017). Perspective: Terahertz science and technology. J Appl Phys.

[5] Geim AK, Grigorieva IV (2013). Van der Waals heterostructures. Nature.

[6] Kuzhir PP, Paddubskaya AG, Volynets NI, Batrakov KG, Kaplas T, Lamberti P, Kotsilkova R, Lambin P (2017). Main principles of passive devices based on graphene and carbon films in microwave—THz frequency range. J Nanophotonics.

[7] Yuan W, Li M, Wen Z, Sun Y, Ruan D, Zhang Z, Chen G, Gao Y (2018). The Fabrication of Large-Area, Uniform Graphene Nanomeshes for High-Speed, Room-Temperature Direct Terahertz Detection. Nanoscale Res Lett.

[8] Xu Z, Wu D, Liu Y, Liu C, Yu Z, Yu L, Ye H (2018). Design of a Tunable Ultra-Broadband Terahertz Absorber Based on Multiple Layers of Graphene Ribbons. Nanoscale Res Lett.

[9] Docherty CJ, Parkinson P, Joyce HJ, Chiu M-H, Chen C-H, Lee M-Y, Li L-J, Herz LM, Johnston MB (2014). Ultrafast transient terahertz conductivity of monolayer MoS2 and WSe2 grown by chemical vapor deposition. ACS Nano.

[10] Huang Y, Zhu L, Yao Z, Zhang L, He C, Zhao Q, Bai J, Xu X (2017). Terahertz Surface Emission from Layered MoS2 Crystal: Competition between Surface Optical Rectification and Surface Photocurrent Surge. J Phys Chem C.

[11] Zhou J, Zhou T, Yang D, Wang Z, Zhang Z, You J, Xu Z, Zheng X, Cheng X-a (2019). Optically Controlled Extraordinary Terahertz Transmission of Bi2 Se 3 Film Modulator. Photon Sensors.

[12] Si K, Huang Y, Zhao Q, Zhu L, Zhang L, Yao Z, Xu X (2018). Terahertz surface emission from layered semiconductor WSe2. Appl Surf Sci.

[13] Yang D-S, Jiang T, Cheng X-A (2017). Optically controlled terahertz modulator by liquid-exfoliated multilayer WS 2 nanosheets. Opt Express.

[14] Valmorra F, Scalari G, Maissen C, Fu W, Schonenberger C, Choi JW, Park HG, Beck M, Faist J (2013). Low-Bias Active Control of Terahertz Waves by Coupling Large-Area CVD Graphene to a Terahertz Metamaterial. Nano Lett.

[15] Tan H, Fan Y, Zhou Y, Chen Q, Xu W, Warner JH (2016). Ultrathin 2D photodetectors utilizing chemical vapor deposition grown WS2 with graphene electrodes. ACS Nano.

[16] Peng L, Jiang X, Li S-m (2018). Multi-functional Device with Switchable Functions of Absorption and Polarization Conversion at Terahertz Range. Nanoscale Res Lett.

[17] Qin H, Sun J, Liang S, Li X, Yang X, He Z, Yu C, Feng Z (2017). Room-temperature, low-impedance and high-sensitivity terahertz direct detector based on bilayer graphene field-effect transistor. Carbon.

[18] Kakenov N, Ergoktas MS, Balci O, Kocabas C (2018). Graphene based terahertz phase modulators. 2D Mater.

[19] Chen Z, Chen X, Tao L, Chen K, Long M, Liu X, Yan K, Stantchev RI, Pickwell-MacPherson E, Xu J-B (2018). Graphene controlled Brewster angle device for ultra broadband terahertz modulation. Nat Commun.

[20] Guo C, Zhang J, Xu W, Liu K, Yuan X, Qin S, Zhu Z (2018). Graphene-Based Perfect Absorption Structures in the Visible to Terahertz Band and Their Optoelectronics Applications. Nanomaterials.

[21] Hafez HA, Kovalev S, Deinert J-C, Mics Z, Green B, Awari N, Chen M, Germanskiy S, Lehnert U, Teichert J (2018). Extremely efficient terahertz high-harmonic generation in graphene by hot Dirac fermions. Nature.

[22] Li J, Zhang T, Chen L (2018). High-Efficiency Plasmonic Third-Harmonic Generation with Graphene on a Silicon Diffractive Grating in Mid-infrared Region. Nanoscale Res Lett.

[23] Zhang L, Huang Y, Zhu L, Yao Z, Zhao Q, Du W, He Y, Xu XPolarized THz Emission from In-Plane Dipoles in Monolayer Tungsten Disulfide by Linear and Circular Optical Rectification. Adv Opt Mater 7. https://onlinelibrary.wiley.com/doi/abs/10.1002/adom.201801314.

[24] Zhang L, Huang Y, Zhao Q, Zhu L, Yao Z, Zhou Y, Du W, Xu X (2017). Terahertz surface emission of d-band electrons from a layered tungsten disulfide crystal by the surface field. Phys Rev B.

[25] Fan Z, Geng Z, Lv X, Su Y, Yang Y, Liu J, Chen H (2017). Optical Controlled Terahertz Modulator Based on Tungsten Disulfide Nanosheet. Sci Rep.

[26] Chen C-Y, Hsieh C-F, Lin Y-F, Pan R-P, Pan C-L (2004). Magnetically tunable room-temperature 2pi liquid crystal terahertz phase shifter. Opt Express.

[27] Zhang H, Guo P, Chen P, Chang S, Yuan J (2009). Liquid-crystal-filled photonic crystal for terahertz switch and filter. JOSA B.

[28] Cunningham PD, Valdes NN, Vallejo FA, Hayden LM, Polishak B, Zhou X-H, Luo J, Jen AK-Y, Williams JC, Twieg RJ (2011). Broadband terahertz characterization of the refractive index and absorption of some important polymeric and organic electro-optic materials. J Appl Phys.

[29] Lippert S, Schneider LM, Renaud D, Kang KN, Ajayi O, Kuhnert J, Halbich M-U, Abdulmunem OM, Lin X, Hassoon K (2017). Influence of the substrate material on the optical properties of tungsten diselenide monolayers. 2D Mater.

[30] Khrapach I, Withers F, Bointon TH, Polyushkin DK, Barnes WL, Russo S, Craciun MF (2012). Novel Highly Conductive and Transparent Graphene-Based Conductors. Adv Mater.

[31] Zhan D, Sun L, Ni ZH, Liu L, Fan XF, Wang Y, Yu T, Lam YM, Huang W, Shen ZX (2010). FeCl3-Based Few-Layer Graphene Intercalation Compounds: Single Linear Dispersion Electronic Band Structure and Strong Charge Transfer Doping. Adv Funct Mater.

[32] Bointon TH, Jones GF, De Sanctis A, Hill-Pearce R, Craciun MF, Russo S (2015). Large-area functionalized CVD graphene for work function matched transparent electrodes. Sci Rep.

[33] Torres Alonso E, Karkera G, Jones GF, Craciun MF, Russo S (2016). Homogeneously Bright, Flexible, and Foldable Lighting Devices with Functionalized Graphene Electrodes. ACS Appl Mater Interfaces.

[34] Bointon TH, Khrapach I, Yakimova R, Shytov AV, Craciun MF, Russo S (2014). Approaching Magnetic Ordering in Graphene Materials by FeCl 3 Intercalation. Nano Lett.

[35] Bezares FJ, Sanctis AD, Saavedra JRM, Woessner A, Alonso-González P, Amenabar I, Chen J, Bointon TH, Dai S, Fogler MM, Basov DN, Hillenbrand R, Craciun MF, García de Abajo FJ, Russo S, Koppens FHL (2017). Intrinsic Plasmon–Phonon Interactions in Highly Doped Graphene: A Near-Field Imaging Study. Nano Lett.

[36] De Sanctis A., Russo S., Craciun M. F., Alexeev A., Barnes M. D., Nagareddy V. K., Wright C. D. (2018). New routes to the functionalization patterning and manufacture of graphene-based materials for biomedical applications. Interface Focus.

[37] Zhukova MO, Grachev YV, Azina LV, Tcypkin AN, Kovalska E, Alonso ET, Russo S, Craciun MF, Baldycheva A, Bespalov VG (2018) Transmission of modified graphene layers on glass, sapphire and polyimide film substrates in UV, visible, NIR and THz spectral ranges In: 2018 International Conference Laser Optics (ICLO), 395–395.. IEEE. 10.1109/LO.2018.8435407. https://ieeexplore.ieee.org/document/8435407/.

[38] De Sanctis A, Jones GF, Wehenkel DJ, Bezares F, Koppens FHL, Craciun MF, Russo S (2017). Extraordinary linear dynamic range in laser-defined functionalized graphene photodetectors. Sci Adv.

[39] De Sanctis A, Barnes MD, Amit I, Craciun MF, Russo S (2017). Functionalised hexagonal-domain graphene for position-sensitive photodetectors. Nanotechnology.

[40] Craciun MF, Bointon TH, Russo S (2015). Is graphene a good transparent electrode for photovoltaics and display applications?. IET Circ Devices Syst.

[41] Wehenkel DJ, Bointon TH, Booth T, Bøggild P, Craciun MF, Russo S (2015). Unforeseen high temperature and humidity stability of FeCl3 intercalated few layer graphene. Sci Rep.

[42] Craciun MF, Khrapach I, Russo S (2013). Properties and applications of chemically functionalized graphene. J Phys Condens Matter.

[43] Hogan BT, Kovalska E, Craciun MF, Baldycheva A (2017). 2D material liquid crystals for optoelectronics and photonics. J Mater Chem C.

[44] Akbari A, Sheath P, Martin ST, Shinde DB, Shaibani M, Banerjee PC, Tkacz R, Bhattacharyya D, Majumder M (2016). Large-area graphene-based nanofiltration membranes by shear alignment of discotic nematic liquid crystals of graphene oxide. Nat Commun.

[45] Jalili R, Aminorroaya-Yamini S, Benedetti TM, Aboutalebi SH, Chao Y, Wallace GG, Officer DL (2016). Processable 2D materials beyond graphene: MoS 2 liquid crystals and fibres. Nanoscale.

[46] Hogan BT, Gromova Y, Kovalska E, Baranov A, Craciun MF, Baldycheva A (2018) Magnetically Tunable Chirality in 2D Liquid Crystalline WS2 Nanocomposites. arXiv preprint arXiv:1804.04745.

[47] Grachev YV, Osipova MO, Kuz’mina AV, Bespalov VG (2014). Determining the working band of frequencies of a pulsed terahertz spectrometer. J Opt Technol.

[48] Balbekin N S, Grachev Ya V, Smirnov S V, Bespalov V G (2015). The versatile terahertz reflection and transmission spectrometer with the location of objects of researches in the horizontal plane. Journal of Physics: Conference Series.

[49] Bespalov VG, Gorodetskii AA, Denisyuk IY, Kozlov SA, Krylov VN, Lukomskii GV, Petrov NV, Putilin SE (2008). Methods of generating superbroadband terahertz pulses with femtosecond lasers. J Opt Technol.

[50] Zhang X, Tan Q-H, Wu J-B, Shi W, Tan P-H (2016). Review on the Raman spectroscopy of different types of layered materials. Nanoscale.

[51] Das A, Chakraborty B, Sood AK (2008). Raman spectroscopy of graphene on different substrates and influence of defects. Bull Mater Sci.

[52] Calizo I, Bao W, Miao F, Lau CN, Balandin AA (2007). The effect of substrates on the Raman spectrum of graphene: Graphene-on-sapphire and graphene-on-glass. Appl Phys Lett.

[53] Yang R, Shi Z, Zhang L, Shi D, Zhang G (2011). Observation of Raman G-Peak Split for Graphene Nanoribbons with Hydrogen-Terminated Zigzag Edges. Nano Lett.

[54] Polat EO, Uzlu HB, Balci O, Kakenov N, Kovalska E, Kocabas C (2016). Graphene-Enabled Optoelectronics on Paper. ACS Photon.

[55] Polat EO, Balci O, Kakenov N, Uzlu HB, Kocabas C, Dahiya R (2015). Synthesis of large area graphene for high performance in flexible optoelectronic devices. Sci Rep.

[56] Docherty CJ, Johnston MB (2012). Terahertz properties of graphene. J Infrared Millimeter Terahertz Waves.

[57] Walowski J, Münzenberg M (2016). Perspective: Ultrafast magnetism and THz spintronics. J Appl Phys.

