# Supplementary material for: Transmission Properties of FeCl3-Intercalated Graphene and WS2 Thin Films for Terahertz Time-Domain Spectroscopy Applications
Source: Nanoscale Res Lett. 2019 Jul 9;14:225. doi: 10.1186/s11671-019-3062-3 (PMC6616562; doi:10.1186/s11671-019-3062-3)
Supplement: Supplementary file 1 — Raman peak positions for different graphene samples on various substrates: SEM and AFM images of few-layer and intercalated few-layer graphene samples. (PDF 550 kb) [file 11671_2019_3062_MOESM1_ESM.pdf]

Transmission Properties of $FeCl_3$ Intercalated Graphene and WS_2 Thin Films for Terahertz Time-Domain Spectroscopy Applications

Supplementary materials

STable 1. Raman peaks position for graphene samples on glass.

Sample	G-peak position, cm^{-1}	2D-peak position, cm^{-1}	I_{2D}/I_G
SLG	1588	2679	1.6
FLG	1582	2711	0.8
i-FLG	1591	2695	1.4
MLG	1582	2720	0.8
i-MLG	1585	2711	0.6

STable 2. Raman peaks position for graphene samples on various substrates.

Substrate	G-peak position, cm^{-1}	2D-peak position, cm^{-1}	I_{2D}/I_G
Kapton	1579	2721	0.8
Glass	1582	2721	0.8
Sapphire	1585, 1612	2703	1.4

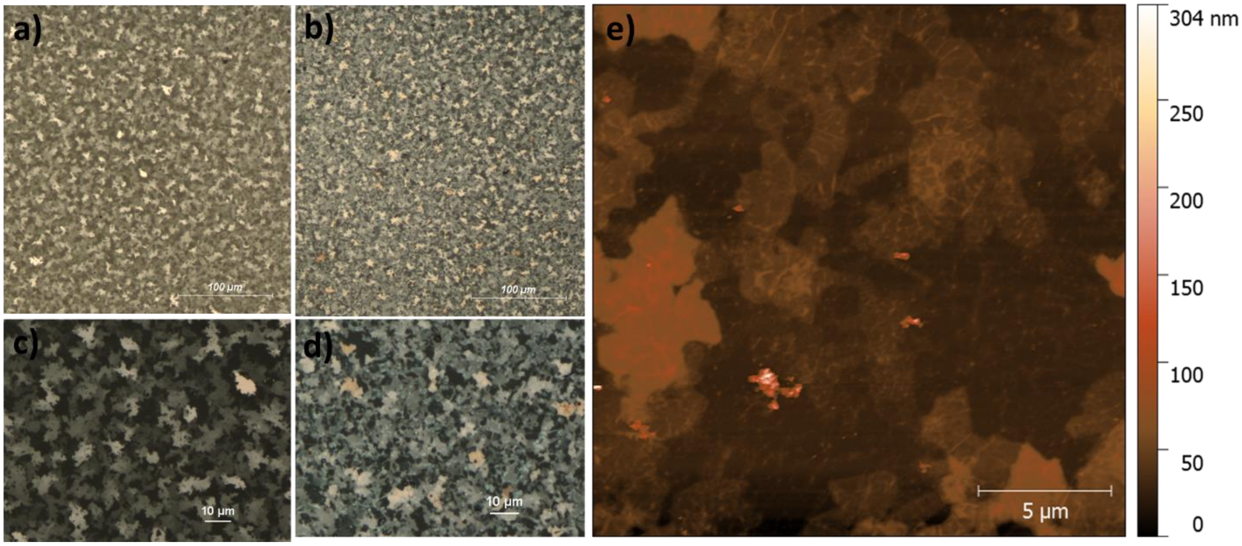

SFigure 1. **a-d)** SEM images of: **a,c)** few-layer graphene, and **b,d)** $FeCl_3$-intercalated few layer graphene. **e)** AFM of $FeCl_3$-intercalated few-layer graphene.
